# Long Term Clinical–Functional and Ultrasound Outcomes in Recreational Athletes after Achilles Tendon Rupture: Ma and Griffith versus Tenolig

**DOI:** 10.3390/medicina57101073

**Published:** 2021-10-08

**Authors:** Carlo Biz, Mariachiara Cerchiaro, Elisa Belluzzi, Nicola Luigi Bragazzi, Giacomo De Guttry, Pietro Ruggieri

**Affiliations:** 1 Orthopedics and Orthopedic Oncology, Department of Surgery, Oncology and Gastroenterology DiSCOG, University of Padova, 35128 Padova, Italy; chiaracerchiaro@gmail.com (M.C.); giacomodeg@hotmail.com (G.D.G.); pietro.ruggieri@unipd.it (P.R.); 2 Musculoskeletal Pathology and Oncology Laboratory, Department of Surgery, Oncology and Gastroenterology, University of Padova, 35128 Padova, Italy; 3 Laboratory for Industrial and Applied Mathematics, Department of Mathematics and Statistics, York University, Toronto, ON M3J 1P3, Canada; robertobragazzi@gmail.com

**Keywords:** Achilles tendon, surgery, ultrasonography, patient outcome assessment, sports, return to sports

## Abstract

*Background and Objectives*: The purpose of this retrospective study was to compare the long-term clinical–functional and ultrasound outcomes of recreational athletes treated with two percutaneous techniques: Ma and Griffith (M&G) and the Tenolig technique (TT). *Materials and Methods*: recreational athletes, between 18 and 50 years of age, affected by acute Achilles tendon rupture (AATR), treated by M&G or Tenolig techniques were recruited. Clinical–functional outcomes were evaluated using Achilles Tendon Rupture Score (ATRS), AOFAS Ankle–Hindfoot score, VAS (for pain and satisfaction) questionnaires, and ultrasound analysis (focal thickening, hypoechoic areas, presence of calcifications, tendinitis and alteration of normal fibrillar architecture). *Results*: 90 patients were included: 50 treated by M&G, 40 by TT. In all, 90% of patients resumed sports activities, with pre-injury levels in 56% of cases after M&G and in 60% after TT. In the M&G group, the averages of the questionnaires were ATRS 90.70 points, AOFAS 91.03, VAS satisfaction 7.08, and VAS pain 1.58. In the TT group: ATRS 90.38 points, AOFAS 90.28, VAS satisfaction 7.76, and VAS pain 1.34. The TT group showed a significantly higher satisfaction and return to sport activities within a shorter time. In the M&G group, ultrasound check showed a significantly greater incidence of thickening and an alteration of fibrillar architecture in the treated tendon. Three infections were reported, including one deep after M&G, two superficial in the TT group, and two re-ruptures in the Tenolig group following a further trauma. *Conclusions*: At long-term follow-up, M&G and TT are both valid techniques for the treatment of AATRs in recreational athletes, achieving comparable clinical–functional results. However, TT seems to have a higher patient satisfaction rate, a faster return to sports and physical activities, and fewer ultrasound signs of tendinitis. Finally, the cost of the device makes this technique more expensive.

## 1. Introduction

The Achilles tendon (AT) is the most frequently injured tendon, accounting for 20% of all large tendon ruptures [1]. AT acute lesions can be defined as a disruption in the conjoined tendon of the gastrocnemius and soleus muscles, usually about 2–6 cm proximal to the tendon insertion into the calcaneus [2]. From an epidemiological perspective, AT ruptures (ATR) exhibit a bimodal age distribution with a first peak in patients between 25 and 40 years old and a second peak in those over 60 years old. Moreover, men are 2 to 12 times more prone to ATR than women [3]. ATR incidence has constantly increased due to aging of the population, growing prevalence of obesity, and increasing practice of sports [4,5]. Acute ruptures of the AT occur most frequently in middle-aged men, an incidence of 69 per 100,000, especially in those participating in sports with energetic repetitive jumping that use eccentric loading and sprinting movements that require pushing-off force [6,7,8].

Further risk factors include pre-existing tendon degeneration, Achilles tendinopathy, systemic corticosteroids, previous steroid injections into or around the Achilles tendon, and use of quinolone antibiotics, among others [9,10].

The diagnosis of ATR is based on clinical tests (Simmonds’ triad: palpation of a gap, Matles test, and calf squeeze test [11]) and confirmed by ultrasound imaging or magnetic resonance image (MRI). Recently, the use of ultrasound imaging has increased in the assessment and management of musculoskeletal pathologies, considering its advantages. Ultrasound imaging is a low-cost technique compared to others, quick to be executed, and a reliable and feasible tool [12]. Moreover, ultrasonography has been used to study foot muscle morphological modifications [13], AT thickness, cross-sectional area, Kager’s fat pad length and gastrocnemius–soleus pennation angle [14] in patients with Achilles tendinopathy.

Despite several randomized controlled trials and meta-analyses investigating surgical versus non-surgical treatment of acute Achilles tendon rupture (AATR), its treatment is still under debate [15,16]: controversy remains about whether nonoperative or operative treatment for AATR is superior, especially for athletic patients [7,17]. The treatment options include conservative treatment, traditional open surgery, mini open techniques, and percutaneous repair [5,18,19]. For elderly patients who have lower functional demands or increased risk of surgical complications, conservative treatment using a short leg resting cast in an equinus position should be suitable [19,20]. However, this treatment is frequently associated with a high rate of tendon re-rupture (up to 20%), tendon elongation, and loss of muscle mass [21]. Numerous surgical procedures have been proposed to repair ATR. Open surgery ensures tendon repair and improves healing, thus leading to a lower re-rupture rate (about 2–5%). However, complications are common, including wound infections, skin tethering, sural nerve damage, and hypertrophic scarring; they have been reported to occur up to 34% of the time [21]. Percutaneous and minimally invasive techniques, such as Ma and Griffith (M&G) or Tenolig using limited incisions, are considered to reduce the risk of complications and seem successful in preventing re-rupture, infection, and nerve lesion, even in athletes [22,23,24,25].

In terms of outcomes, resuming sports activity to preinjury levels after ATR is often the primary goal of the patients. However, a systematic review reported that 20% of patients included in the review did not return to sports [26].

To the best of our knowledge, there is only one study in the literature comparing the short- to mid-term outcomes of these two percutaneous techniques in the general population, showing no differences among the two procedures in term of clinical–functional results and return to daily life activities [27]. The aim of the present study was to investigate and compare long term clinical, functional, and ultrasound outcomes of a consecutive series of recreational athletes affected by AATR and treated by M&G or Tenolig percutaneous techniques at our institution. Our hypothesis was that the two techniques are comparable to each other in terms of restoration of optimal length, early rehabilitation, and return to daily and sports activities.

## 2. Materials and Methods

### 2.1. Patients

This study was designed as a retrospective, single-center, comparative, clinical, and ultrasound study including a consecutive series of Caucasian patients affected by a midportion ATR and treated at our level-I healthcare trauma center (Orthopedics and Orthopedic Oncology Department, University-Hospital of Padova) from June 2010 to May 2014 by one of the following percutaneous surgical procedures: (a) Ma and Griffith (M&G) technique or (b) Tenolig technique (TT).

At the time of the clinical, functional, and ultrasound evaluation, all subjects participating in this long-term follow-up study received a thorough explanation of the risks and benefits of inclusion and gave their oral and written informed consent to publish the data. The Local Ethics Committee of Padova approval was obtained prior to any data collection (Prot. n. 0031087, Padova 3/26/2021). The study was performed in accordance with the ethical standards of the 1964 Declaration of Helsinki as revised in 2013 and conducted ethically according to the most recent international standard [28].

### 2.2. Inclusion and Exclusion Criteria

The inclusion criteria were the diagnosis of ATR with at least a 5 mm gap at the rupture site confirmed by ultrasound. As reported by clinical notes at time of visit, all eligible patients had the Simmonds triad for diagnosis confirmation, which includes (1) an altered angle of declination of the foot or “angle of dangle” referring to the loss of tension in an ATR, which causes a more dorsiflexed position of the injured foot [29] (or Matles test: sensitivity of 0.88 and positive predictive value 0.92 [30]); (2) a gap felt on palpation (sensitivity of 0.73 and the positive predictive value 0.82 [30]); and (3) the most popular Thomson’s calf squeeze test [31]. Additionally, all patients had ultrasound at diagnosis.

Further inclusion criteria were (1) recreational athletes (at least 2 training sessions per week) between 18 and 50 years old, (2) a complete mid-portion ATR, typically located at 2–7 cm from the insertion onto the calcaneus [32], (3) ATR repair surgery within 2 weeks after trauma, (4) Body Mass Index (BMI) between 18 and 25, and (5) American Society of Anesthesiologists (ASA) class globally estimated surgical risk 1 or 2.

Exclusion criteria were (1) patients affected by inveterate ATR, (2) AT re-rupture, (3) incomplete injuries, (4) open or bilateral lesions, (5) patients affected by metabolic diseases, (6) insertional or myotendinous junction injury, (7) gait problems or stiffness in the tibiotarsal joint prior to trauma and previous surgery, (8) degenerative rupture without significant trauma, and (9) percutaneous surgery after 14 days from the injury.

**Figure 1:**
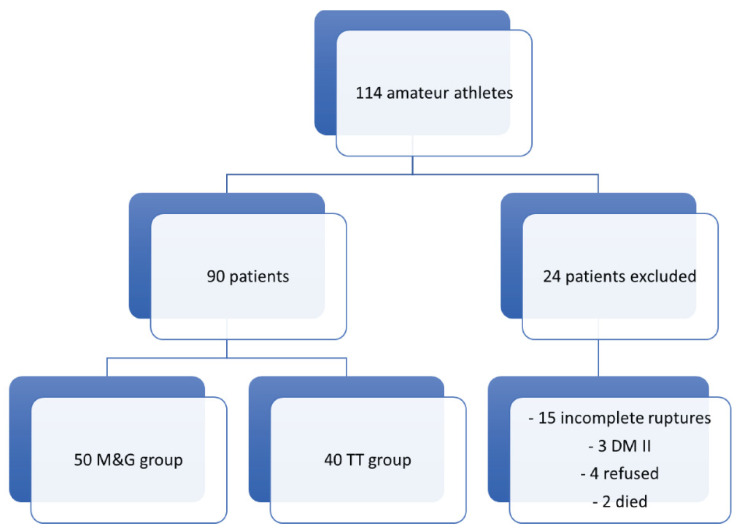
Flowchart of patient selection. DM II: diabetes mellitus type II; M&G: Ma and Griffith technique; TT: Tenolig technique.

During a 4-year period of enrolment, 114 recreational athletes were operated at our institution for acute ATR using M&G or TT. Twenty-four patients were excluded: 15 had incomplete rupture, 3 had type II diabetes, 4 refused to participate, and 2 were deceased at the time of analysis. Therefore, a final cohort of 90 patients was enrolled (Figure 1).

### 2.3. Surgical Percutaneous Techniques

All surgical procedures were performed by the same team of 2 surgeons (the senior authors) with a similar learning curve required for both techniques and considerable experience in minimally invasive orthopedic surgery. As reported previously [28], this Foot and Ankle team had operated more than 60 patients using these techniques before 2010. During the study period (2010–2014), both procedures were chosen without any technique preference by the same team and alternated every month depending on the availability of the materials in the surgical theater.

For all procedures, after regional anesthesia, patients were positioned prone on the operating table, and no calf tourniquet was applied.

#### 2.3.1. Ma and Griffith Technique (M&G)

**Figure 2:**
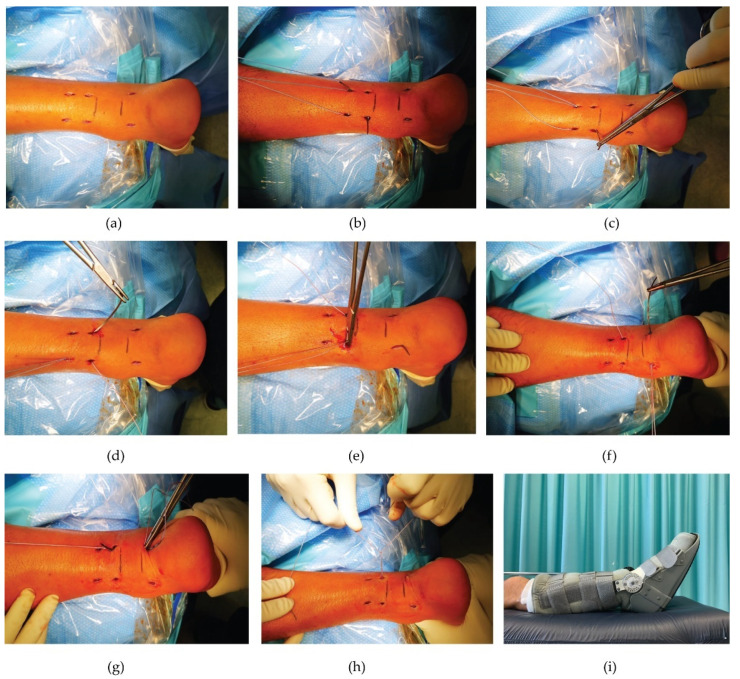
Ma and Griffith surgical technique. (**a**) During surgery, the site of the ruptured tendon is marked. (**b**) Six small incisions (5 mm) are performed at the sides of the proximal (4) and distal stump (2) of the tendon (2 cm apart from each other) to be able to direct and cross the wire suture (**c**) using nonabsorbable suture woven through the proximal and distal parts of the tendon. (**d**–**f**) The suture, with two semicurved needles at the ends, is transversely passed through the tendon followed by a (diagonal) cross-suture (at each end of the thread) in proximal to distal direction. (**g**,**h**) Maintaining the ankle in maximum equinus position, the suture is tied after arming the stumps and cutting the needles off, making the two segments of the tendon adhere. (**i**) Immediately after surgery, a brace in equinus position is applied.

This percutaneous suture was performed as originally described by G. Ma and T. Griffith in 1977 [22], using nonabsorbable suture (Tycron 2) woven through the proximal and distal parts of the tendon (Figure 2). Briefly, 6 or 8 small incisions (less than 1 cm), according to gap extension, were made on the sides of the proximal and distal stumps of the tendon, about 2 cm proximal and distal to the rupture. Then, the suture, with two curved cutting needles at the end, was brought out through an enlarged medial incision of 1 cm. With the ankle in maximum equinus position, the suture was tied after arming the tendon stumps and cutting the needles, bringing the tendon ends into apposition.

#### 2.3.2. Tenolig Technique (TT)

**Figure 3:**
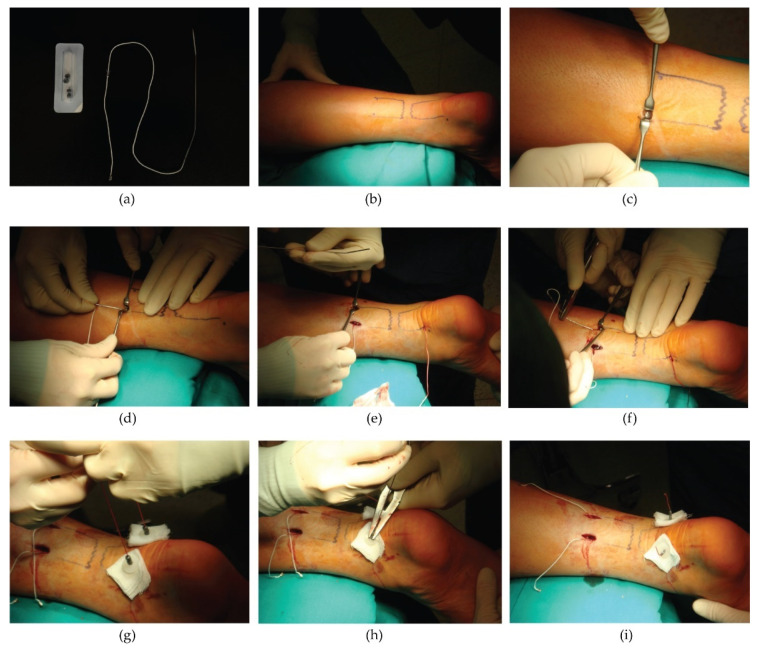
The Tenolig surgical technique. (**a**) The Tenolig device and its components. (**b**) During surgery, the tendon rupture point is marked. (**c**) The first small skin incision (<1 cm) is performed approximately 6 cm above the rupture zone. (**d**) The first needle is inserted taking care to allow the anchor to penetrate perpendicularly into the proximal side of the tendon and then remove it from the distal portion of the tendon, 4 or 5 cm below the rupture point. (**e**) The second incision is made, and (**f**) the same procedure is repeated with the second needle. At the end, both needles are cut. (**g**) The plastic buttons are applied to protect the skin. The two straps are pulled tight simultaneously while the ankle is maintained in equinus position, (**h**) and the weights are threaded to fix the straps distally. (**i**) The sutures of the skin are left long to allow the removal of the implant 45 days after surgery.

The Tenolig^®^ device (FH Orthopedics, Heimsbrunn, France) consists of a Dacron thread with a diameter of 0.85 mm and a length of 36 cm on which a harpoon of 7 mm in diameter is mounted; the thread has a needle at its end with a flexible triangular tip 15 cm long and an anchoring system that is mounted after passing through the distal tendon stump outside the skin (Figure 3). A small skin incision is made on the medial or lateral side at the level of the proximal portion of the tendon, about 6 cm above the rupture area, to prevent the proximal fixation system from meeting already malacic tissue. The needle is then inserted, taking care to penetrate the proximal and distal portion of the tendon, and is made to come out to the sides of the calcaneal tuberosity. The procedure is repeated on the opposite side of the same tendon with an additional Tenolig^®^ device. Both threads are then pulled using the proximal harpoon in direct contact with the tendon. The suture threads are stretched with the ankle in maximum equinus position, and the distal anchoring system is then applied.

### 2.4. Post-Operative Protocol of Both Procedures

All patients followed the same postoperative protocol, with the exception of the outpatient procedure for the removal of the devices for those operated by the TT. After surgical medication, a bandage to maintain the ankle in equinus position was applied. The same day of the surgery, an articulated brace was applied before patients’ discharge maintaining the ankle blocked in maximum equinus position for 3 weeks (1st–21st day). Patients could walk with crutches without loading on the operated leg. Then, for the following 3 weeks (22nd–45th day), a progressive reduction of the equinus position was allowed, progressive load as tolerated maintaining the brace during walking, carrying it at 90° of ankle flexion progressively, engaging in active and passive physiotherapy, and removing the brace only at rest. The Tenolig® device and brace were removed 45 days after surgery. The proximal harpoons were removed by two small incisions under local anesthesia on the previous surgical incisions. From the 45th to the 90th day, the use of shoes with an insole on the heel, complete load, active and passive physiotherapy, avoidance of jumps but including range of motion, stretching and nonimpact strengthening exercises were suggested. This post-operative rehabilitation program was performed with the assistance of different physiotherapists, who adopted similar re-education procedures for both patient groups, including kinesthetic and proprioceptive training by using the balance control platform [33]. Finally, the insoles previously applied were removed, and sports activities were allowed 90 days after surgery, while competitive sports activities after 120 days.

### 2.5. Patient Assessment

From March to May 2021, data collection, as well as radiological and clinical evaluation, was retrospectively performed by two independent investigators, not directly involved in the patients’ operative treatment, who identified patients treated for ATR by searching hospital patient records. Patients’ characteristics (gender, age at trauma, comorbidities, pre-trauma corticosteroid use and smoking habits), trauma characteristics (affected side, type of sport, mechanism of injury, concomitant injuries, presence of pre-trauma tendinopathy), treatment, and post-operative characteristics (type and timing of treatment, complications) were collected from the hospital's electronic database for every patient included. According to the repair method used, the patients were divided into two groups:

I° group: Ma and Griffith technique (M&G);

II° group: Tenolig technique (TT).

### 2.6. Clinical and Functional Outcome Measures

During the follow-up orthopedic evaluation, carried out from March to May 2021 at our hospital outpatient center, the following objective clinical tests were performed on each patient: Thompson test, Matles test, gap felt on palpation, bilateral single heel rise test, bilateral measurement of the calf circumference 10 cm distal to the tibial tuberosity, and difference in degrees of dorsiflexion and plantarflexion between the operated leg and the contralateral leg. At the follow-up visit, to assess the quality of life following surgery, the following questionnaires were administered to all patients: Achilles tendon Rupture Score (ATRS) [34,35] and American Orthopedic Foot and Ankle Society ankle–hindfoot score (AOFAS) [36,37]. The ATRS questionnaire is a highly reliable, valid, and sensitive tool for measuring symptom- and physical-activity-related outcomes of patients who have undergone Achilles tendon repair surgery. It consists of 10 questions, each worth a maximum of 10 points per question. AOFAS includes 9 questions related to pain (1 question; 40 points), function (7 questions; 50 points), and alignment (1 question; 10 points). A score of 90–100 is considered an excellent result, 75–89 good, 50–74 as sufficient, and less than 49 points is considered a failure or a bad result. Further, a 0–10 visual analogue scale (VAS) was used to quantify patient satisfaction of the results, where 0 means maximum dissatisfaction and 10 full satisfaction. The duration of physiotherapy (weeks), the return to work and post-surgery sports (weeks), and the return to pre-injury sports (yes, no, partial) were recorded.

### 2.7. Ultrasound Outcome Measures

During the same follow-up clinical and functional evaluation, ultrasound outcome measures were performed. With the subject prone on the examination table, the heels protruding from the table, and the ankles flexed 90°, both ATs (normal and operated) were fully evaluated in the longitudinal and axial planes. The echostructural continuity of the tendon, the presence of hypoechoic areas, the presence of calcifications, and the presence/absence of inhomogeneity of the fibrillar architecture of the tendons were investigated. Tendon abnormalities in scans were classified as focal thickening measured in mm, hypoechoic areas (present/absent), presence of calcifications (present/absent), tendinitis (present/absent), and alteration of normal fibrillar architecture (present/absent).

### 2.8. Complications

Complications were divided between minor and major. Minor complications included wound complications, pain, swelling, and weakness. Major complications included deep infection, chronic infection, deep vein thrombosis, sural nerve-related complaints, pulmonary embolism, tendon shortening, and tendon lengthening.

### 2.9. Statistical Analysis

The a priori power analysis was conducted using the software G*Power 3.1.9.7 for Windows. The minimum sample size required was computed based on our working hypothesis and a previously published study [27], which found an OR of 0.17 ((95%CI 0.00–1.97), *p* = 0.23) for M&G compared with Tenolig. Parameters were selected as follows: an alpha error probability of 0.05 and a power of 0.80–0.95. The minimum sample size varied from 36 to 57 subjects.

Before handling the data, they were pre-processed and visually inspected for quality control, missing data, and potential outliers. Normality of data distribution was verified conducting the Shapiro–Wilk test, which was preferred to other tests (including the Kolmogorov–Smirnov or the D’Agostino–Pearson omnibus test) given the small sample size employed. Continuous variables were expressed as mean standard deviation, while categorical parameters were computed as percentages where appropriate. Uni-(Student’s *t*-test for unpaired, independent samples, chi-squared test) and multivariate (logistic regression model) analyses were conducted to capture eventual differences between the two surgical techniques. For all analyses, figures with p values less than or equal to 0.05 were considered statistically significant. All statistical analyses were performed using the commercial software “Statistical Package for Social Sciences” (SPSS version 24.0 for Windows, IBM, Armonk, NY, USA.

## 3. Results

### 3.1. Patient Data

Baseline characteristics of the enrolled patients are depicted in Table 1.

The mean follow-up was 9.7 years (range 6.8–10.6 years). Overall, the patients were satisfied and had high values of ATRS and AOFAS score (Video S1–S4).

### 3.2. Demographic, Clinical, and Functional Outcomes between M&G and T Group

The M&G group characteristics are described in Table 2. In all, 48% of them reported a tendon injury during a sports activity (soccer, basketball, tennis, running); most involved in the rupture was soccer (26%), followed by basketball (6%).

Patient comorbidities and risk factors were recorded as well: 5 patients were smokers, and 2 had suffered from contralateral ATR; 39 patients (78%) reported no symptoms of pre-rupture tendinopathy; 11 patients (22%) reported tendinopathy in the year preceding the rupture. The mean duration of post-operative physiotherapy performed was 3.88 weeks.

The TT group characteristics are reported in Table 2. In total, 52.5% reported tendon injury during the sporting activity; most involved in the rupture was soccer (22.5%), followed by martial arts (5%). The left and right sides were affected equally. Patient comorbidities and risk factors were recorded as well: four patients were smokers and one patient had undergone systemic corticosteroid therapy a month before the break. Thirty-six patients (90%) reported that they had no symptoms of pre-rupture tendinopathy, while four patients (10%) reported that they had tendinopathy in the year prior to the rupture. The mean duration of post-operative physiotherapy performed was 3.38 weeks.

No statistically significant differences were reported between the two groups regarding age of the patients, gender, the prevalence of the affected side, the presence of tendinopathy on the affected side in the year preceding the rupture, duration of the physiotherapy cycle, and return to work (Table 2). However, a difference between the two groups was reported for satisfaction of the surgical approach and the return to sport and physical activity. Satisfaction was higher in the TT group compared to the M&G group (*p* = 0.035). The patients operated with the Tenolig device returned to sports and physical activity earlier compared to the M&G group (*p* = 0.003).

The Thompson test and the Matles test, index of continuity of the AT, were bilaterally negative in all patients of both groups, as well as sign of the gap.

In the M&G group, the single heel rise test on the operated side was weakly positive in 10%, positive in 6%, and weakly positive in 4% on the healthy side.

In the T group, the single heel rise test of the operated side was weakly positive in 15%, never completely positive, and weakly positive in 5% on the healthy side.

The mean calf circumference difference was found to be smaller in the TT group (2.40 cm) compared to the M&G group (3.30 cm) (*p* < 0.0001).

In the M&G group, the average of the results of the ATRS questionnaire was between good and excellent. The average of the AOFAS questionnaire was 91.03 points, 62% of patients obtaining a result between 90 and100, which is considered excellent, and 38% obtaining a result between 75 and 89, considered good.

In the TT group, the results of the ATRS questionnaire were good to excellent, with 65% of patients obtaining a result between 90 and 100 and 35% a result between 75 and 89. The results of the AOFAS questionnaire were good to excellent, with 60% of patients achieving a result between 90 and 100 and 40% achieving a result between 75 and 89. VAS for pain indicated the absence of pain in both groups. No statistically significant differences emerged for the ATRS score, AOFAS score, time to return to work, and return to pre-injury sports level (Table 2).

### 3.3. Ultrasound Outcomes between M&G and T Group

In the M&G group, the mean diameter of the operated tendon was higher compared to that in the TT group (Table 3) (*p* < 0.0001).

No differences were observed regarding areas of hypoechogenicity between the two groups (*p* > 0.05). Intratendinous calcifications were found in a higher number of M&G patients compared to TT patients (*p* > 0.05).

A higher number of M&G patients presented tendinitis compared to TT patients (*p* = 0.0430). The normal fibrillar architecture of the tendon appeared altered in a higher number of the M&G patients compared to T patients (*p* = 0.0013).

### 3.4. Complications

In the M&G group, five patients (10%) reported complications: three minor and two major. The minor complications included one patient who had significant ankle edema such as to limit physiotherapy and walking for 2 months, and two who patients had superficial surgical wound infections resolved with antibiotic therapy. Regarding the major complications, one patient had ankle stiffness (about 15° less dorsal and plantarflexion than the contralateral ankle) due to tendon shortening that did not regress after several physiotherapy sessions, and one patient had a deep wound infection with dehiscence resolved by two surgical cleansings and Linezolid therapy.

In the TT group, five patients (12.5%) reported only minor complications: two patients had superficial infections with dehiscence resolved by antibiotic therapy, two patients underwent re-rupture of the AT but due to other trauma after 2 months from surgery, and one patient had a mobilization of one of the two anchors of the Tenolig after 8 days, but the post-operative protocol was successfully completed with good functional results.

### 3.5. Multivariate Logistic Regression Analysis

Multivariate logistic regression analysis confirmed the results obtained by the univariate analysis (Table 4). Patients treated with TT returned to perform sports and physical activity earlier (*p* = 0.0012). Calf circumference difference (*p* = 0.008) and echographic diameters of operated tendons (*p* = 0.003) were smaller. They reported lower occurrence of tendinitis (*p* = 0.0436) and abnormal fibrillar architecture (*p* = 0.0020).

## 4. Discussion

Despite numerous high-evidence studies, the optimal management of the ATR, conservative or operative, continues to be a subject of debate within the orthopedic community [7]; the superiority of one type of procedure over the other has not yet been demonstrated by comparing the results of the different procedures described in the literature: open, minimally invasive, or percutaneous [38]. Currently, operative treatment is particularly recommended in active patients who require a rapid return to daily activities, including sports. However, surgery is expensive and presents a higher complication rate compared to conservative therapy [38].

Based on our experience and on the literature, conservative treatment is not advisable for professional athletes and even for recreational ones due to the higher re-rupture rates and worse clinical and functional outcomes [39,40,41]. Percutaneous repair represents a good compromise in non-elite level athletes with respect to open surgery, as it allows the tendon stumps to be brought together with lower rates of complications in wound healing compared to other techniques. Moreover, percutaneous procedures better preserve the vascularity of the paratenon and its sliding surface in comparison with more invasive techniques [42].

Although functional and ultrasound outcomes have been reported in several studies [43,44], to the best of our knowledge, only a few studies have measured ATRS more than 7 years after rupture [45,46], and none comparing these two percutaneous techniques, M&G and TT, in recreational athletes affected by acute ATR.

The most important finding of this long follow-up comparative analysis was that the post-operative scores (AOFAS, ATRS, and VAS for intervention satisfaction) reached good to excellent levels even at long-term follow-up with adequate patient satisfaction in both groups, and all patients, regardless of the technique used, regained normal walking. Patient satisfaction rate (VAS) was higher in group TT, probably due to a significantly earlier return to sports activity. Return to work was earlier in the TT group, even if the difference between the two techniques was not statistically significant (*p* = 0.141). As for the Ma and Griffith technique, the results regarding return to work were better than those in other studies published in the literature; this is probably because the patients in our study were young recreational athletes (39.68 ± 7.95). Karabinas et al. reported a return to work after 9 weeks in 19 patients treated with Ma and Griffith’s technique, versus an average of 5 in our study [47]. Rouvillain et al. reported a return to work of about 12 weeks [48]. Taglialavoro et al. reported 14.2 weeks for the M&G technique and 12.5 weeks for the TT technique. However, it should be noted that these patients were not recreational athletes [27].

Overall, more than 70% of the 90 recreational athletes with AATR returned to their previous sports activity level after a standardized nonoperative early full weightbearing treatment protocol in this retrospective observational study. The return to sports activities was statistically significantly earlier in the TT group (26.08 weeks). However, Jallageas et al. reported an earlier return (after 18.5 weeks) with TT [49], while Zayni et al. using the M&G technique reported a return after an average of 32.30 weeks [50], comparable to our results. Rouvillain et al. reported a return after 21.4 weeks using the M&G technique [48]. In all, 90% of patients in both groups returned to the same pre-injury physical activity level, 56% in the M&G group and 60% in the T group. These results are in line with those reported in the literature [47,48,49,50].

Although our study involves recreational athletes that have fewer functional demands and require less rehabilitation assistance compared to professional athletes, the results obtained do not differ greatly from the studies by Grassi et al. and Jack et al., demonstrating the validity of the percutaneous techniques in returning to sports, at least at a recreational level [51,52]. The first study reported that 96% of professional athletes returned to sports after surgical repair, 82% at the same pre-injury level, and the second described less than 75% of NFL professional athletes returned to pre-injury levels [51,52]. However, in none of these studies was the return to sports activity in relation to a specific surgical technique analyzed.

In our study, the post-operative scores (AOFAS, ATRS, and VAS for pain and intervention satisfaction) reached excellent/good levels, with adequate patient satisfaction, and all patients, regardless of the technique used, regained normal walking. The VAS of the satisfaction of the intervention was higher and statistically significant (*p* = 0.035) in group TT, probably due to an earlier return to work and sports activity. The average of the ATRS and AOFAS score was similar between the M&G group and the TT group. The value of AOFAS was lower than that of other studies. In Lacoste et al., who analyzed intraoperative ultrasonography during Tenolig^®^ repair, the average AOFAS score was 95 and ATRS was 91.3 [25]. In Jallageas et al., the average AOFAS score was 94 [49]. Zellers et al. highlighted that the study of the AT morphology by ultrasound in the follow-up (first 6 months post-surgery) is useful for assessing healing and the prognosis of function in the following months [53]. Our study confirms the advisability of an ultrasound evaluation even at a longer follow-up.

The calf circumference difference was significantly lower in the TT group compared to the M&G group. Jallageas et al. compared the open technique with the TT in 31 sports patients observing a difference of 13 mm between the two calves in the group operated with the percutaneous technique, which was less than the mean difference reported in our study [49].

Regarding complications, there were 3 infections in the M&G group, one of which was deep, and two superficial in the TT group, in line with the literature reporting 0.6% deep infections in contrast to 3.6% with open techniques [54]. No sural nerve (SN) injuries, transient or permanent, were recorded in our sample, compared to generally 5.5% reported in the literature after minimally invasive surgery and 6.4% after Tenolig repair, which decrees to 2.6% when the SN is visualized by US [18,25,55].

This excellent result can be explained by the fact that for the M&G group, the final knot was always performed on the medial side of the ankle, while for the T group, the lateral profile of the tendon is carefully respected without ever going beyond it, following palpatory insertion of the harpoon inside the tendon. Instead, there were two re-ruptures in group T after 3 months but following excessive trauma and in any case in line with the literature, which reports a rate of 3.7–6% in percutaneous surgery and 2.8–3% in open surgery [56]. The comparison of complications between the two groups in our study is not statistically significant.

Areas of hypoechogenicity were found in 56% (M&G group) and 47.5% (TT group) of the operated tendons, which is in contrast with the study by Bleakney et al. [44]. In this study, areas of hypoechogenicity were found in 23.6% of patients treated by the M&G technique [44]. Since only percutaneous repairs were performed compared to the Bleakney study, these areas are probably due to the suture thread or to small tendon discontinuities next to the rupture. This is supported also by the presence of calcifications found in both groups (22% M&G and 17.5% TT), which might be present before the injury (in the case of sports patients) and not a reaction to the surgical insult [44].

The M&G group showed a higher percentage of patients with tendinitis compared to the TT group. Moreover, alterations of normal fibrillar architecture were found in a higher percentage of the M&G group compared to the T group. These echographic differences support a superiority of the TT over the M&G technique. The difference found, 64% (M&G group) and 42.5% (TT group), may represent a subclinical tendinopathy predisposing to rupture or could be a consequence of altered walking and altered load on the healthy limb following surgery. Gigante et al. had already compared this parameter between the open and percutaneous technique (Tenolig), showing a difference between the two techniques [57].

The open and the percutaneous techniques are both safe and effective in repairing the ruptured AT, and both afford the same degree of restoration of clinical, ultrasound, and isokinetic patterns [57]. Nevertheless, the main advantages of percutaneous repair include reduced cutaneous complications, use of analgesics, and operation times; day surgery procedures; fewer deep infections; faster recovery, enhancing overall patient compliance with improved of AOFAS scores; and lower healthcare costs [41,55,58,59,60]. As Ma and Griffith and Tenolig satisfied most of these advantages in our patient groups, another implication of this study is that they can be considered preferable to open repair in managing subcutaneous ruptures of AT also in non-professional sports practicing adults, confirming the results of previous reports [57,61].

Although the present study did not clearly answer what is the best treatment for AATRs in recreational athletes, our data suggest that both percutaneous techniques did not have negative implications in the two groups analyzed, such as the most frequent complications reported in the literature after surgical management: re-rupture attributable to the incorrect technique and sural nerve lesion [54,59]. However, insufficiency of the gastrocnemius–Achilles tendon complex was observed only in a single patient of the T group due to imperfect stump juxtaposition that did not enable restoration of original tendon length. Further, in accordance with previous authors [54,57], all our operations were performed in day surgery with an incremental relationship between costs and benefits. However, the cost of Tenolig (almost 1000 euros) made this procedure more expensive than M&G.

Finally, future randomized controlled clinical trials with a larger sample size are necessary (1) to compare the two percutaneous techniques to open techniques and/or non-operative treatment methods to provide further useful information for foot and ankle surgeons in the management of AATR in amateur athletes; and (2) to test their effectiveness also in professional ones and chronic ruptures.

### Strengths and Weaknesses

The strengths of our study include (1) the standardization of operative procedures, and postoperative protocol including aftercare, according to our institutional protocol in use at our institution since 2010 [27], for both percutaneous techniques performed by the same team of two senior surgeons; this aspect avoids confounding bias and allows adequate methodology for comparative reasons; (2) the long follow-up; (3) the fact that this was a comparative study with an adequate number of patients in both groups for statistical analysis; (4) the analysis of the clinical outcomes, carried out separately by independent investigators; (5) the multivariable statistical analysis performed by an independent statistician.

We are also aware of the study’s weaknesses: (1) single center, case series, and retrospective design, (2) lack of randomization, although the patients were operated during the 4-year study period using one or the other procedure depending on the monthly availability of the materials in the surgical theater and without any technique preference by the surgeons; (3) absence of an open technique group, and/or a non-operative control group, which prevented us from further comparing results; and (4) no MRI or instrumented gait analysis having been routinely performed during post-operative treatment or at last follow-up.

## 5. Conclusions

In accordance with the initial research hypothesis, this retrospective and comparative study demonstrated that the two percutaneous techniques, M&G and TT, achieved similar long clinical–functional results in the operative management of AATRs that occurred in recreational athletes. However, at long follow-up, the patients of the TT group seemed to show a higher patient satisfaction rate, a faster return to sports and physical activities, and fewer ultrasound signs of tendinitis or abnormal fibrillar architecture of operated AT compared to patients treated with the M&G technique.

For these reasons, it is the opinion of the authors that the TT may be preferable in the operative treatment of AATRs in recreational athletes under 50 years old, even if the cost of the device must be considered carefully.

## Figures and Tables

**Table 1:** Demographics, clinical outcomes of the cohort (*n* = 90).

Parameter	Value
Age (years), mean ± SD	40.90 ± 7.59
Gender, number (%)	
Male	74 (82.2%)
Female	16 (17.8%)
Affected Side, number (%)	
Right	50 (55.6%)
Left	40 (44.4%)
Tendinopathy	15 (16.7%)
FKT (weeks), mean ± SD	3.65 ± 1.80
Follow-up (years), mean ± SD	9.7 ± 2.4
Satisfaction of intervention, mean ± SD	7.46 ± 1.54
Return to work (weeks), mean ± SD	4.80 ± 2.64
Return to sports and physical activity (weeks), mean ± SD	28.84 ± 10.20
Return to pre-injury physical activity, number (%)	
No	9 (10.0%)
Partially	29 (32.2%)
Yes	52 (57.8%)
Heel rise test (operated side)	14 (15.6%)
Heel rise test (healthy test)	4 (4.4%)
ROM, mean ± SD	−2.21 ± 4.22
VAS score, mean ± SD	1.44 ± 0.75
ATRS score, mean ± SD	90.52 ± 3.53
AOFAS score, mean ± SD	90.61 ± 4.27
Calf circumference difference (cm)	2.89 ± 1.08
Echographic diameter of operated tendon (mm)	14.26 ± 1.78
Echographic hypoechogenicity (operated side)	47 (52.2%)
Echographic calcifications (operated side)	18 (20.0%)
Echo-Doppler tendinitis	49 (54.4%)
Echographic abnormal fibrillar architecture	66 (73.3%)
Complications	10 (11.1%)

SD = standard deviation, ROM = range of motion, VAS = visual analogue scale, ATRS = Achilles tendon Total Rupture Score, AOFAS = The American Orthopedic Foot and Ankle Society score.

**Table 2:** Comparison of demographics and clinical outcomes between the M&G and T group.

Parameter	Intervention		*p*-Value
	M&G (50 patients)	TT (40 patients)	
Age, mean ± SD	39.68 ± 7.95	41.88 ± 7.22	0.172
Gender, number (%)			0.951
Male	41 (82.0%)	33 (82.5%)	
Female	9 (18.0%)	7 (17.5%)	
Affected Side, number (%)			0.345
Right	30 (60.0%)	20 (50.0%)	
Left	20 (40.0%)	20 (50.0%)	
Tendinopathy	11 (22.0%)	4 (10.0%)	0.121
FKT (weeks), Mean ± SD	3.88 ± 1.86	3.38 ± 1.72	0.193
Satisfaction of intervention, mean ± SD	7.08 ± 1.75	7.76 ± 1.29	0.035
Return to work (weeks), mean ± SD	5.03 ± 2.79	4.62 ± 2.52	0.472
Return to sports and physical activity (weeks), mean ± SD	32.30 ± 9.43	26.08 ± 10.03	0.003
Return to pre-injury physical activity, number (%)			0.918
No	5 (10.0%)	4 (10.0%)	
Partially	17 (34.0%)	12 (30.0%)	
Yes	28 (56.0%)	24 (60.0%)	
Heel rise test (operated side)	8 (16.0%)	6 (15.0%)	0.897
Heel rise test (healthy test)	2 (4.0%)	2 (5.0%)	0.820
ROM, mean ± SD	−2.64 ± 4.23	−1.68 ± 4.20	0.283
VAS score, mean ± SD	1.58 ± 0.75	1.34 ± 0.75	0.141
ATRS score, mean ± SD	90.70 ± 3.55	90.38 ± 3.54	0.671
AOFAS score, mean ± SD	91.03 ± 4.71	90.28 ± 3.90	0.414
Calf circumference difference (cm)	3.30 ± 1.02	2.40 ± 0.96	<0.0001
Complications	5 (10.0%)	5 (12.5%)	0.709

**Table 3:** Comparison of ultrasound outcomes between the M&G and T group.

Parameter	Intervention		*p*-Value
	M&G (50 patients)	TT (40 patients)	
Echographic diameter of operated tendon (mm)	14.97 ± 1.90	13.39 ± 1.16	<0.0001
Echographic hypoechogenicity (operated side)	28 (56.0%)	19 (47.5%)	0.425
Presence of calcifications (operated side)	11 (22.0%)	7 (17.5%)	0.598
Echo-Doppler tendinitis	32 (64.0%)	17 (42.5%)	0.043
Echographic abnormal fibrillar architecture	43 (86.0%)	23 (57.5%)	0.001

**Table 4:** Multivariate logistic regression analysis of co-variates associated with Tenolig surgical technique.

Parameter	Regression Coefficient	Standard Error	Wald Coefficient	OR	95%CI	*p*-Value
Return to sports/physical activity	1.50	0.47	10.44	4.50	1.81−11.21	0.001
Calf circumference difference	1.24	0.47	7.07	3.45	1.39−8.61	0.008
Echographic diameter of operated tendon	1.32	0.45	8.56	3.75	1.55−9.09	0.003
Echo-Doppler Tendinitis	−0.88	0.44	4.07	0.42	0.18−0.98	0.044
Abnormal fibrillar architecture	−1.67	0.54	9.51	0.19	0.07−0.54	0.002

## Data Availability

The dataset supporting the conclusions of this review is available at our institution.

## Supplementary Materials

The following are available online at https://www.mdpi.com/article/10.3390/medicina57101073/s1. The four videos demonstrate the complete healing of the Achilles tendons after percutaneous suture according to the Ma and Griffith (videos S1 and S2: left ankle) and Tenolig techniques (videos S3 and S4: right ankle): full flexion–extension of the ankle with respect to the contralateral (videos S1 and S3) and normal gait of operated patients (S2 and S4).
